# Construct validity for the self-reported competency and sub-construct associated characteristics of Romanian physicians in autism spectrum disorder

**DOI:** 10.1186/s12909-021-02999-9

**Published:** 2021-11-20

**Authors:** Mohammad H. Rahbar, Iuliana Dobrescu, Shezeen Gillani, Manouchehr Hessabi, Sori Kim, Mihaela Stancu, Florina Rad

**Affiliations:** 1 Division of Clinical and Translational Sciences, Department of Internal Medicine, McGovern Medical School, The University of Texas Health Science Center at Houston, 6410 Fannin Street, Suite 1100.05, UT Professional Building, Houston, TX 77030 USA; 2 Biostatistics/Epidemiology/Research Design (BERD) core, Center for Clinical and Translational Sciences (CCTS), The University of Texas Health Science Center at Houston, 6410 Fannin Street, Suite 1100.05, UT Professional Building, Houston, TX 77030 USA; 3 Department of Epidemiology, Human Genetics, and Environmental Sciences, School of Public Health, The University of Texas Health Science Center at Houston, 1200 Pressler Street, Houston, TX 77030 USA; 4 Child and Adolescent Psychiatry Department, University of Medicine and Pharmacy “Carol Davila”, Bucharest, Romania; 5 Child and Adolescent Psychiatry, “Prof. Dr.Alex. Obregia” Psychiatry Hospital, Bucharest, Romania; 6 Department of Biostatistics & Data Science, School of Public Health, The University of Texas Health Science Center at Houston, Houston, TX USA

**Keywords:** Construct validity, Factor analysis, Competency of physicians, Autism Spectrum Disorder (ASD), Romania

## Abstract

**Background:**

Lack of physicians’ knowledge regarding mental health, including Autism Spectrum Disorder (ASD) could have adverse effects on affected individuals’ health and quality of life. The purpose of this study was to provide construct validity for a modified questionnaire in order to determine the self-reported competency for underlying sub-constructs in ASD, make inferences on perceived competence in ASD based on a sample of Romanian physicians, and identify physicians’ characteristics associated with these sub-domains of competency.

**Methods:**

For this survey, we modified a questionnaire that was used in Pakistan and Turkey, and administered it to a sample of 383 practicing physicians in Romania to assess their perceived competency regarding ASD. Exploratory factor analysis on 12 knowledge questions revealed five sub-domains: stigma, potential causes, children’s behavior, misconceptions, and educational needs associated with ASD knowledge. Using General Linear Models, we determined physicians’ characteristics that predict the total competency score and various competency sub-scores.

**Results:**

Seventy-five percent of the responding physicians were female and 30% had over 30 years practicing medicine. The majority (73–94%) of physicians have correctly responded to some basic questions regarding knowledge about ASD. We also found that younger physicians were more knowledgeable about potential causes of ASD than older physicians (Adjusted Mean Score (AMS): 2.90 vs. 2.18, *P* < 0.01), while older physicians knew more about the behavior of children with ASD (AMS: 0.64 vs. 0.37, *P* = 0.02). We found a significant interaction (*P* < 0.01) between television as source of ASD knowledge and city where the clinic is located in relation to knowledge of the physicians regarding stigma related to ASD. However, the total score was not associated with the variables associated with sub-domains.

**Conclusion:**

Using factor analysis, we demonstrated construct validity of five sub-domains related to Romanian physicians’ knowledge about ASD that include stigma, potential causes, behavior in ASD children, special education needs, and misconceptions related to ASD. The lack of significant association of the knowledge of physicians on ASD neither with the Psychiatry nor the Pediatric ward rotations at medical school may support the need for improving the curriculum on ASD in Romanian medical schools.

**Supplementary Information:**

The online version contains supplementary material available at 10.1186/s12909-021-02999-9.

## Background

Knowledge, attitude, and practice (KAP) surveys are commonly used for assessment and improvement of competencies in medical education [1], and public health programs in different countries [1, 2]. Specifically, an inadequate level of general knowledge of physicians regarding mental health issues such as Autism Spectrum Disorder (ASD) could have adverse effects on patients’ health and quality of life in various ways [3–5]. Furthermore, the lack of general knowledge of physicians about ASD could further delay its diagnosis [6] and initiation of timely and appropriate cost-effective interventions, particularly in communities in which mental health is considered a stigma, and communities where physicians may hold a potential implicit bias regarding their patient’s mental health concerns [7, 8]. Moreover, greater focus is needed to educate a new generation of motivated medical school students about mental disorders including ASD [9, 10], who will be able to influence the perceptions of general populations about these disorders, and to further develop their interests in pursuing related opportunities to seek formal residency and fellowship trainings in Psychiatry and other related fields. However, currently the medical education systems in many countries lack sufficient focus on ASD and other related disorders. There are some instances where physicians have only heard of mental health issues such as ASD through the media [10].

Several studies from different countries have assessed the knowledge, attitude, and practices of physicians and health workers regarding ASD with different tools and methods of evaluation. For example, a study from Turkey used a questionnaire with open-ended questions [11]; some studies from Pakistan [12, 13] and Turkey [14] used Likert-scale questions; a study from Nepal used focus groups and semi-structured interviews [15]; and another from Nigeria used multiple choice questions where the correct choice for each question received a score of one (1), while the other two incorrect choices received a score of zero [16]. All of these studies noted a deficiency in physicians’ competency regarding ASD. Studies from Pakistan [12, 13] reported a significant degree of misconceptions regarding ASD among general practitioners and physicians. In addition, Minhas et al. highlighted a high degree of stigma, lack of available services, and the limited levels of ASD knowledge in general physicians in Pakistan and India, which takes a significant toll on parents, and reduces their faith in the medical professional’s competence and ability to help children with ASD [17]. This study also noted that most physicians specializing in ASD and other mental health conditions stay within urban areas, leaving rural populations with little to no support [17]. Some studies indicated a lack of formal training regarding ASD and the management of diagnosed children in terms of the available options [18, 19].

Often, knowledge of healthcare professionals, including physicians, is assessed via questionnaires without any established construct validity. The common methodology weakness for the aforementioned studies is the use of a total score (or percentage of a specific response) to represent ASD knowledge among physicians and practitioners, without identifying underlying sub-constructs for the total score, nor providing evidence for validity of the construct for the overall knowledge score, or the knowledge sub-scores [12–14, 16]. However, more recent studies have reported information on psychometric assessments and internal consistency [20, 21]. Shawahna et al. (2021) assessed adequacy of medical training for students in Palestine to provide care for individuals with ASD through a multicenter cross-sectional study by establishing test-retest reliability for the sub-constructs that includes the medical students’ familiarity, knowledge, confidence, and willingness to learn about ASD in general [21].

Typically, exploratory factor analysis is used to identify latent structures and underlying sub-constructs for the total score. Without performing factor analysis on all the questions used to calculate the total score, there may be a situation where the independent latent structures (domains) are not identified, resulting in a total score that does not fully represent these sub-constructs. Though a previous study has used factor analysis for a similar assessment [10], to the best of our knowledge, this is the first study that has assessed both additive and interactive associations of various factors with knowledge, attitude, and practices of physicians regarding ASD, with a particular focus on data from Romania.

Romania is an ethnically diverse, upper-middle-income country in Eastern Europe [22, 23]. A significant proportion (90.4%) of Romanian children who require psychiatric care initially visit a general physician for advice and guidance [24]. Some physicians in Romania utilize many of the instruments that are used internationally for the assessment of ASD, however there are some instruments that are not adapted to Romanian cultures and customs [25]. For example, the Checklist for Autism in Toddlers (CHAT) [26] and its newer versions are not available for assessment of ASD in Romania [25], but are used internationally to assess a broad range of developmental disorders including ASD in children under 3 years of age. In 2011, the Romanian Health Ministry developed the Screening Questionnaire for Autism Spectrum Disorder [Chestionarul de Screening pentru Tulburări de Spectru Autist (CS-TSA)] modeled after the CHAT to specifically aid Romanian physicians in the early detection of ASD between the ages of 0–3 years [27]. Since 2016, CS-TSA has been included in the subsidized services of the National Health Insurance Company, and it has become mandatory for family practitioners to administer the questionnaire. The Romanian curriculum for residents in family medicine has not changed significantly since 1996 [28]. The current postgraduate curriculum for family physicians requires 1 month of psychiatric training during the residency program, however, there is no focus on child and adolescent psychiatry in the training program (https://rezidentiat.ms.ro/curricule/2017/medicina_de_familie_2017.pdf). Post graduates specializing in pediatric care are required to have a three-month child psychiatry rotation during their residency training program (https://rezidentiat.ms.ro/curricule/2018/pediatrie_2017.pdf) (A Google Translation of part of this document is provided as a Supplementary Material). During the 6 years of medical school, students only have one-week rotation of child and adolescent psychiatry. Even though the medical school curriculum regarding ASD hasn’t changed in the past decade, access to more ASD cases, a greater awareness of the disorder in physicians, and greater access to knowledge disseminated from other researchers and media (as a potential source for general knowledge about ASD in general) may have contributed to an increase in ASD knowledge of Romanian physicians with pediatric psychiatry training.

In this cross-sectional study we aim to 1) provide evidence for the validity of a modified tool to determine the self-reported competency, both in the overall and underlying domains/sub-constructs of knowledge related to ASD; 2) make inferences on overall perceived competence in ASD of a sample of Romanian family physicians; and 3) identify a series of physician characteristics that predict the various sub-domains of competency, either independently or interactively.

## Methods

### Study design

For this survey, investigators at the University of Texas Health Science Center at Houston (UTHealth) and “Carol Davila” University of Medicine and Pharmacy (UMF) collaborated to develop a questionnaire by modifying a previously established questionnaire used in Pakistan [13] and Turkey [14], to assess the knowledge, attitude, and practices of physicians regarding ASD in Romania. The questionnaire was translated to Romanian and back translated to English by the Romania team to ensure the accuracy of the translations. The study protocol was approved by the Institutional Review Boards (IRBs) of UTHealth (HSC-GEN-15-0844) and Ethical Review Committee (ERC) (PO-35-F-03) of Carol Davila-UMF.

### Participants

Participants in this survey were selected from a listing of all the private practices and clinics of physicians in Romania. A volunteer team that included medical students from the “Professor Alexandru Obregia” Clinical Psychiatry Hospital were provided the aforementioned list to assist with distribution and collection of completed questionnaires from the physicians at each of their practices from January to July in 2017. As a result of this effort, they collected a total of 383 completed questionnaires.

### Data management and quality assurance of data

The UTHealth team provided a Research Electronic Data Capture (REDCap) [29] bilingual database in Romanian and English for data entry from the questionnaires. We used the double-data entry method [30] to minimize discrepant data as part of our quality assurance procedures. Initially, the Carol Davila team in Romania entered data from the questionnaires into the REDCap database. Then the completed questionnaires were sent to the UTHealth team in the US for a second round of data entry. Since physicians responded to the open-ended questions in Romanian, we identified a graduate student at UTHealth, who is a native Romanian speaker, to translate the responses into English, which were then entered into the REDCap database.

### Instruments

#### The questionnaire for assessing knowledge, attitude, and practices of physicians about ASD

The Questionnaire contained three sections. Section A had 19 questions focused on assessing socioeconomic and demographic information including age and sex. This section also inquired about the participant’s background in the medical field and their current practice (e.g., location of clinic, number of patients seen in a day in their practice, etc.). This section ended by asking if the physician personally knew someone with ASD.

Section B had six questions to assess the participants’ knowledge and attitude about ASD. This section first asked if the participant had ever heard of “*Autism or Autism Spectrum Disorder*.” If the participant marked “*Yes*,” then in the following question they were asked to provide the sources where they heard about ASD. All participants were then asked to provide an estimate for the prevalence of ASD in Romania, the US, and globally. The physicians were subsequently asked that out of every 100 children that s/he sees in her/his practice, how many children have ASD. At the end of this section there were14 statements that were prepared to assess various aspects of the knowledge, attitude, and practices of the Romanian physicians about ASD, for which the response options were in a 5-point Likert-scale: “*Strongly agree*,” “*Agree*,” “*Undecided*,” “*Disagree*,” and “*Strongly disagree*.”

For developing an overall score to quantify various aspects of physicians’ knowledge about ASD, we have classified the Likert-scale responses to binary. Specifically, depending on whether the statement in the question was true or false, the “*Undecided*” groups were merged either with the “*Agree*” or “*Disagree*” groups. For example, since question 2, “*Autism is a possible result of neglect by the parents*,” is false, the physicians who marked “*Undecided*” for question 2 were merged with the “*Agree*” and “*Strongly agree*” groups. Conversely, because question 8, “*Children with autism require special education*,” is true, the physicians who marked “*Undecided*” for question 8 were merged with the “*Disagree*” and “*Strongly disagree*” groups. As a result of this reclassification, we derived binary variables based on whether the statements in the question were true or false. Since questions 1, 6, 8, 9, 10, 11, and 12 are true, we recoded “*Strongly agree*” and “*Agree*” as 1, and “*Undecided*”, “*Disagree*” and “*Strongly disagree*” as 0. For questions 2, 3, 4, 5, 7, and 14, which are false, “*Strongly agree*”, “*Agree*”, and “*Undecided*” were recoded as 0, and “*Disagree*” and “*Strongly disagree*” were recoded as 1. We determined that the responses to Question 13, “*Parents in Romania tend to think their children are at risk for autism*,” may not be reliable because the prevalence or risk of ASD in Romania is currently unknown. Additionally, we determined that the responses to Question 9, “*Children with autism deliberately misbehave*,” may not be reliable as they could fluctuate depending on how the physicians interpreted the statement. Due to their ambiguous nature, Questions 9 and 13 were excluded from further analyses. Based on the remaining 12 questions, we calculated the sum of all the question scores as a total score for *physicians’ knowledge about ASD*, which we used for further analyses.

Section C had six questions designed to assess the participants’ practices about ASD. This section assessed the physician’s knowledge of available tools to screen children for ASD, and to list the screening tools they had used in the past, if any. The physicians were then asked about early indicators of ASD in 2-year-old children, and what they do when they suspect a child has ASD. Section C continued by asking the physicians about which ASD diagnostic tools they had used in the past (“*Have you ever used any of the following to diagnose a child with autism or Autism Spectrum Disorder*”). Next the physicians were presented with a 4-point Likert-scale question (“*In diagnosing children with autism, the following symptoms are*”) comprised of 11 accurate or inaccurate symptoms used to diagnose ASD with response options of “*Necessary*,” “*Not necessary, but helpful*,” “*Not helpful*,” and “*Don’t know*.” The last question in this section asked physicians to provide their opinion on ways to reduce the prevalence of ASD in Romania.

#### Sample size justification and statistical power

In order to estimate the proportion of physicians who are knowledgeable about ASD within a 5% margin of error with 95% confidence, we needed to survey at least 384 physicians. Considering that around 5% of the surveys were expected to be incomplete, we planned to survey 400 physicians. In our survey, a total of 383 physicians completed the survey. This sample size is also sufficient to detect an effect size of 0.3 or greater with at least 80% power at a 5% level of significance, assuming the ratio of physicians in the two groups compared (e.g., male vs. female physicians) with respect to their knowledge sub-score ranged from 0.40 to 0.60.

### Statistical analysis

#### Descriptive analysis

We used descriptive statistics to summarize various characteristics of the study population, including age and gender of the participants. Some of the open-ended questions were categorized before analysis. Physicians’ age was categorized as a dichotomous variable with categories of ≤ 35 & > 35 years. Since we received a variety of responses for names of the medical schools where the physicians earned their degrees, these responses were reduced to three categories: 1) Carol Davila University of Medicine and Pharmacy, 2) Grigore *T. Popa* University of Medicine and Pharmacy, and 3) Other Medical Schools. The physicians that responded to the survey had clinics in various cities of Romania, and the responses were categorized based on the number of survey responses received for each city, leaving us with four categories: Bucharest, Suçeava, Brăila, and other cities. We also asked physicians to write the year in which they completed their most recent continuing education course, for which the responses were divided into before 2007, between 2007 and 2014, and after 2014. The physicians were also asked to write down how many years they had been practicing medicine, and the responses were categorized as more than 30 years, between 16 and 30 years, and 15 years or less.

The responses for the average number of patients each physician saw in his/her practice daily and the average number of patients under the age of 12 seen by each physician in a day were categorized as: ≤ 20 patients, 21–40 patients, and > 40 patients per day. The self-reported responses for the average time a physician spent at his/her practice each day were categorized based on an 8-h workday (≤ 8 h and > 8 h per day). Likewise, responses for the average time a physician spent with each patient was classified as ≤15 min and > 15 min. Only binary (yes or no) responses were allowed for other variables such as whether the physician completed specific ward rotations in or after medical school, and if the physicians had heard of ASD in the past, and were reported as such.

All physicians were asked whether they had heard of ASD in the past. If a physician left this question blank, we interpreted that the physician did not know, hence included this in the group that have not heard of ASD. Physicians were then asked to mark where they had heard of ASD as their source of ASD knowledge (SAK). The 10 SAK options provided for selection were: medical school, continuing education, conferences, primary literature, colleagues, television, newspaper, internet, radio, and other sources not included in the list. Physicians were allowed to choose more than one SAK. Responses to the SAK question from physicians that had indicated that they had not heard of ASD or did not respond to the question were included in the analysis as an additional category. This resulted in a three-level variable for each SAK: those who heard of ASD from the selected SAK (Yes); those who heard of ASD but did not hear of ASD from this SAK (No); and those who had not heard of ASD.

#### Developing sub-scores to quantify various domains of physicians’ knowledge about ASD

To investigate the possible involvement of independent sub-domains in the overall knowledge score based on the 12-statement Likert-scale questions, we performed Exploratory Factor Analysis to determine these latent factors [31]. We used the criteria specified in Samanic, et al. [31] for extracting variables for various factors. That is: Eigenvalue of 1.00 (i.e., % of variance explained by each factor is equivalent to the variance explained by only one variable) or more were retained for the analysis. Questions with an absolute factor loading value of ≥ 0.40 were included in the composite score (weighted score or factor score) for further analysis. The Factor Analysis resulted in five composite scores with factor-like sub-scores derived from questions included in each factor, with equal weights of 1.

#### Analysis of total score and sub-scores of physicians’ knowledge about ASD

The total score and factor-like sub-scores were used as dependent variables in univariable and multivariable General Linear Models (GLMs) or linear regression models to determine the physicians’ characteristics that were associated with their ASD knowledge sub-scores. Demographic, socioeconomic, and current clinical practice of the physicians were treated as categorical variables and were considered independent variables in the GLMs. Based on results from the univariable GLMs, we selected independent variables with a *P-*value ≤ 0.05 to include in the multivariable GLMs. We also explored possible interactions of these variables with each other in relation to the knowledge total score and sub-scores using GLMs. Variables were considered significantly associated with the total score and sub-scores if they had a *P*-value ≤ 0.05. Each of the final GLMs for the total score and each sub-score were used to obtain and report the Least Square Means (LSMeans) as the adjusted mean sub-scores (AMS), after adjusting for other variables in their final respective models. All analyses were performed using the SAS 9.4 [32] statistical software package.

## Results

Descriptive analysis of demographic and socioeconomic characteristics from the 383 questionnaires revealed that about 75% of the participating physicians who responded were female, and more than 80% were over the age of 30. Though all participants graduated from universities in Romania, more than half of the physicians graduated from UMFs (54.3%). Majority of the physicians completed the following rotations during their medical school years: Family medicine (65.8%); Pediatrics (89.3%); and Psychiatry (78.1%). Most participants practiced medicine within the capital city of Bucharest (57.2%) with patients mainly coming from urban areas (72.5%). More than 60% of physicians had practiced medicine for more than 15 years at the time of this survey (Table 1).

**Table 1:** Descriptive characteristics of physicians that participated in the survey (N = 383)

Characteristics	Categories			N (%*)
ender ^a^	Male			85 (22.2)
	Female			288 (75.2)
Age ^b^	≤ 35 years old (Y/O)			50 (13.1)
	> 35 Y/O			320 (83.6)
Attended medical school in Romania ^c^	Yes			379 (99.0)
Type of Medical school attended ^d^	Private			13 (3.4)
	Public			361 (94.3)
Medical school of degree conferral ^e^	Carol Davila University of Medicine & Pharmacy			208 (54.3)
	Grigore *T. Popa* University of Medicine & Pharmacy			64 (16.7)
	Other			94 (24.5)
Ward rotations completed in medical school	Family Medicine	Yes		252 (65.8)
		No		131 (34.2)
	Surgical	Yes		308 (80.4)
		No		75 (19.6)
	OB/GYN	Yes		316 (82.5)
		No		67 (17.5)
	Psychiatry	Yes		299 (78.1)
		No		84 (21.9)
	Pediatrics	Yes		342 (89.3)
		No		41 (10.7)
	Internal Medicine	Yes		340 (88.8)
		No		43 (11.2)
	Geriatrics	Yes		120 (31.3)
		No		263 (68.7)
	Emergency	Yes		193 (50.4)
		No		190 (49.6)
	Other	Yes		121 (31.6)
		No		262 (68.4)
Training after medical school ^**^	Training at Certificate level			355 (92.7)
	Training at Masters level			39 (10.2)
	Training at Doctorate level			28 (7.3)
	Other levels of training			107 (27.9)
	No training			34 (8.9)
Most recent continuing education completed at the time of survey ^f^	> 2014			159 (41.5)
	2007–2014			76 (19.8)
	< 2007			73 (19.1)
Years practicing medicine ^g^	≤ 15 Years			97 (25.3)
	16–30 Years			151 (39.4)
	> 30 Years			115 (30.0)
City in which the physician’s clinic is located ^h^	Bucharest			211 (55.1)
	Suçeava			23 (6.0)
	Brăila			16 (4.2)
	Other			119 (31.1)
Average number of patients seen by the physician in a day ^i^	> 40 patients			16 (4.2)
	21–40 patients			176 (46.0)
	0–20 patients			166 (43.3)
Where the physician’s patients come from? ^j^	Urban			270 (70.5)
	Rural			46 (12)
	Both urban and rural			56 (14.6)
Has the physician heard of autism? ^k^	Yes			305 (79.6)
	No			72 (18.8)
	**Physician’s sources of knowledge about ASD** **(*n*** **= 305)**	Medical School	Yes	137 (44.9)
			No	168 (55.1)
		Continuing Education	Yes	166 (54.4)
			No	139 (45.6)
		Conferences	Yes	225 (73.8)
			No	80 (26.2)
		Primary Literature	Yes	205 (67.2)
			No	100 (32.8)
		Colleagues	Yes	168 (55.1)
			No	137 (44.9)
		Television	Yes	145 (47.5)
			No	160 (52.5)
		Newspaper	Yes	75 (24.6)
			No	230 (75.4)
		Internet	Yes	230 (75.4)
			No	75 (24.6)
		Radio	Yes	27 (8.9)
			No	278 (91.1)
		Other sources ^l^	Yes	37 (12.1)
			No	253 (83.0)

*Row percentages may not add up to 100% due to missing data; ** It is possible for physicians to have participated in multiple training programs after medical school, as a result, the percentages do not add up to 100%. The number of missing data for the variable with ^a^ are 10; for the variable with ^b^ are 13; for the variable with ^c^ are 4; for the variable with ^d^ are 9; for the variable with ^e^ are 17; for the variable with ^f^ are 75; for the variable with ^g^ are 20; for the variable with ^h^ are 14; for the variable with ^i^ are 25; for the variable with ^j^ are 11; for the variable with ^k^ are 6; and for the variable with ^l^ are 15

The Factor Analysis revealed five factors. The factor loadings are provided in Table 2, though for the sake of generalizability to other settings, we decided to use equal weights of 1 in calculating the factor-like scores. As shown in Table 2, Factor 1–5 represent physicians’ knowledge of: 1) stigma related to ASD, 2) potential cause (s) of ASD, 3) behavior of children with ASD, 4) misconceptions about ASD, and 5) educational needs related to ASD. The total score is the sum of scores for the 12 questions used for the factor analysis. Details on the five sub-scores, and total score used for the subsequent analyses are listed and described in Table 2.

**Table 2:** Distribution of physicians’ responses to several statements used for assessing knowledge, attitude, and practices of physicians in Romania about Autism Spectrum Disorder (ASD) and information about the five sub-scores and the total score representing physicians’ knowledge about ASD in Romania identified based on Factor Analysis applied to physicians’ responses (N= 383)

Questions or statements *^†^	Statement is True (T) or False (F)	Frequency of physicians N (%**)		Latent characteristics of variables involved in the Factor and Factor-like sub-score
		Agree	Disagree	
1. Children with autism are often detached from their family and peers. ^a^	T	280 (73.1)	96 (25.1)	Factor 1: Physicians’ knowledge of stigma related to ASD **Sub-score 1 = Q10 + Q11 + Q12**
2. Autism is a possible result of neglect by the parents. ^b^	F	160 (41.8)	217 (56.7)	
3. Autism is a precursor for schizophrenia. ^a^	F	175 (45.7)	201 (52.5)	
4. It is often difficult to distinguish between autism and schizophrenia. ^c^	F	215 (56.1)	160 (41.8)	Factor 2: Physicians’ knowledge of potential cause (s) of ASD **Sub-score 2 = Q2 + Q3 + Q4 + Q5**
5. Autism is more prevalent in higher socioeconomic classes. ^c^	F	191 (49.9)	184 (48)	
6. Children with autism are not affectionate. ^b^	T	179 (46.7)	198 (51.7)	
7. Children can grow out of autism. ^b^	F	353 (92.2)	24 (6.3)	Factor 3: Physicians’ knowledge of the behavior of children with ASD **Sub-score 3 = Q1 + Q6**
8. Children with autism require special education. ^d^	T	361 (94.3)	18 (4.7)	
10. There is a stigma against autism in my community. ^e^	T	191 (49.9)	188 (49.1)	Factor 4: Physicians’ knowledge of misconceptions about ASD **Sub-score 4 = Q7 + Q14**
11. Diagnosing a child with autism will lead to discrimination against this child and his/her family. ͩ	T	239 (62.4)	137 (35.8)	
12. In general, there is a negative opinion toward children diagnosed with autism. ^a^	T	189 (49.4)	189 (49.4)	
14. Autism is preventable ^e^	F	160 (41.8)	217 (56.7)	Factor 5: Physicians’ knowledge of educational needs related to ASD **Sub-score 5 = Q8**
Total score based on the 12 questions used in Factor Analysis				**Total score** = **Q1 + Q2 + Q3 + Q4 + Q5 + Q6+ Q7+ Q8+ Q10 + Q11+ Q12+ Q14**

*Participants were asked, “indicate your response to each of the following statements”. ^**†**^ Questions 9 and 13 were dropped from the analysis due to their ambiguity. ** Row percentages may not add up to 100% due to missing data; for ^a^ there are 7 missing data; for ^b^ there are 6; for ^c^ there are 8; for ^d^ there are 4; and for ^e^ there are 5

The relative frequency of responses to the 12 questions indicated that, of the 383 physicians surveyed, more than 70% of physicians correctly agreed with the statement that “*Children with autism are often detached from their family and peers*.” More than half of the physicians (56.7%) also correctly disagreed with the statement “*Autism is a possible result of neglect by the parents*.” In addition, a majority of physicians (52.5%) correctly disagreed with the statement “*Autism is a precursor for schizophrenia*.” However, most of the physicians (56.1%) incorrectly agreed with the statement “*It is often difficult to distinguish between autism and schizophrenia*.” Surprisingly, more than 90% of physicians agreed with the incorrect statement “*Children can grow out of autism*.” On the other hand, nearly 60% of physicians agreed with the statement “*There is a stigma against autism in my community*,” and 62.4% of physicians agreed that “*In general, there is a negative opinion toward children diagnosed with autism*.” Details regarding the knowledge of physicians about ASD are provided in Table 2.

When asked about essential symptoms sought for diagnosing ASD, 88.3% of physicians correctly reported that “*impaired social interaction*” was necessary for a diagnosis. Additionally, 87.0% of physicians identified “*impaired communication*” as a necessary symptom for diagnosis, and 73.1% also recognized “*lack of eye contact*” as an essential symptom. More than 60% of physicians acknowledged that “*language disturbances*”, and “*restricted and repetitive behavior*” were also crucial symptoms to look for when assessing a child for ASD. More than half of the respondents stated that “*visual hallucinations*” (52.5%), “*confirmed schizophrenia*” (51.7%), and “*hearing voices*” (50.4%) were not helpful symptoms for an ASD diagnosis. Only 9.6% of physicians considered “*depression*” as a necessary symptom when considering an ASD diagnosis. Further details on knowledge of the physicians about ASD symptoms are displayed in Table 3.

**Table 3:** Knowledge of the physicians about ASD symptoms (N = 383)*

Symptoms	Number and Percent of physicians			
	Necessary n (%)	Not Necessary but Helpful n (%)	Not Helpful n (%)	Don’t Know n (%)
Impaired social interaction ^a^	332 (88.3)	27(7.2)	3 (0.8)	14 (3.7)
Impaired communication ^b^	320 (87.0)	45 (12.2)	3 (0.8)	0 (0.0)
Visual hallucinations ^c^	13 (3.5)	85 (22.7)	197 (52.5)	80 (21.3)
Restricted and repetitive behavior ^c^	247 (65.9)	80 (21.3)	12 (3.2)	36 (9.6)
Lack of eye contact ^a^	275 (73.1)	54 (14.4)	13 (3.5)	34 (9.0)
Confirmed schizophrenia ^c^	10 (2.7)	68 (18.1)	194 (51.7)	103 (27.5)
Hearing voices ^d^	15 (4.0)	84 (22.5)	188 (50.4)	86 (23.1)
Language disturbances ^a^	229 (60.9)	111 (29.5)	15 (4.0)	21 (5.6)
Hypersensitivities to certain environments ^a^	172 (45.7)	113 (30.1)	23 (6.1)	68 (18.1)
Depression ^e^	36 (9.6)	117 (31.3)	99 (26.5)	122 (32.6)
Anxiety ^c^	87 (23.2)	132 (35.2)	59 (15.7)	97 (25.9)

*Participants were asked “in diagnosing children with autism spectrum disorder (ASD), the following symptoms are …”; ^a^ Data are missing for 7; ^b^ Data are missing for 15b Data are missing for 15

^c^ Data are missing for 8 physicians; ^d^ Data are missing for 10; ^e^ Data are missing for 9

Findings from the factor analysis revealed that sub-scores for Factor 1 mainly depended on the physicians’ responses to the following three statements: “*There is a stigma against autism in my community*,” “*Diagnosing a child with autism will lead to discrimination against this child and his/her family*,” and “*In general, there is a negative opinion toward children diagnosed with autism*” (True = 1 or False = 0). About half of the physicians (43.7%) inaccurately responded to at least two of the three variables. Factor 2 sub-scores were based on the physicians’ responses to these four statements: “*Autism is a possible result of neglect by the parents*,” “*Autism is a precursor for schizophrenia*,” “*It is often difficult to distinguish between autism and schizophrenia*,” “*Autism is more prevalent in higher socioeconomic classes*” (True = 1 or False = 0). Only 16.3% (*n* = 61) of physicians responded accurately to all four of these statements. The sub-scores for Factor 3 comprised of physicians’ responses to the following two statements: “*Children with autism are often detached from their family and peers*,” and “*Children with autism are not affectionate*” (True = 1 or False = 0). Of the physicians who responded to these questions, 57.9% responded incorrectly to at least one of the statements. Sub-scores for Factor 4 depended on the physicians’ responses to two statements, “*Children can grow out of autism*,” and “*Autism is preventable*” (True = 1 or False = 0), that are commonly held misconceptions related to ASD. It is thought that ASD has a multifactorial etiology with a strong genetic component, and is manageable, but not preventable, nor curable. Only 4.2% of physicians responded accurately to both of the Factor 4 statements. Factor 5 was dependent on the physicians’ response to the statement, “*Children with autism require special education*” (True =1 or False = 0), and 94.3% (*n* = 361) of physicians agreed with this statement. The ranges for each sub-score are reported in Table 4. The mean of the total score, which combines scores for all 12 questions used for factor analysis, was 6.5, with a standard deviation of 2.0. The total score ranged from 1 out of 12 to 11 out of 12, with a first quartile (Q1) value of 5, a median (Q2) of 6, and a third quartile (Q3) value of 8.

**Table 4:** Latent sub-scores for physicians’ knowledge, attitude, and practices as determined by factor analysis and their sub-scores ( N = 383)

Factors	Latent characteristic of variables involved in the Factor	Min Score	Max Score	Frequency of physicians at sub-scores for each Factor Row % (n)				
				0	1	2	3	4
**Factor1**^**a**^	Knowledge of stigma related to ASD (***N*** **= 376**)	0	3	26.1 (98)	17.6 (66)	13.3 (50)	43.1 (162)	NP^*^
**Factor2** ^**b**^	Knowledge of potential cause(s) of ASD (***N*** **= 374**)	0	4	15.2 (57)	20.3 (76)	27.5 (103)	20.6 (77)	16.3 (61)
**Factor3** ^**c**^	Knowledge of the behavior of children with ASD (***N*** **= 375**)	0	2	20.3 (76)	37.6 (141)	42.1 (158)	NP	NP
**Factor4** ^**d**^	Misconceptions about ASD (***N*** **= 377**)	0	2	47.8 (180)	48.0 (181)	4.2 (16)	NP	NP
**Factor5** ^**e**^	Knowledge of educational needs and outcomes related to ASD (***N*** **= 379**)	0	1	4.7 (18)	94.3 (361)	NP	NP	NP
**Total** ^f^	Total Score based on the 12 questions used in Factor Analysis (***N*** **= 371**)	1	11	Mean = 6.5	SD^*^ = 2.0	Q1^*^ = 5	Q2^*^ = 6	Q3^*^ = 8

^*^ Standard Deviation (SD), First quartile (Q1), median (Q2), third quartile (Q3)

^a^ Factor 1 sub scores were missing for 7 physicians; ^b^ Factor 2 sub scores were missing for 9 physicians; ^c^ Factor 3 sub scores were missing for 8 physicians; ^d^ Factor 4 sub scores were missing for 6 physicians; ^e^ Factor 5 sub scores were missing for 4 physicians; ^f^ Total score was missing for 12 physicians; ^*^ NP = Not possible due to maximum score limitation

### Characteristics associated with physicians’ knowledge of stigma related to ASD (factor 1)

Our multivariable GLM with the “physicians’ knowledge about the existing stigma related to ASD in the Romanian community” sub-score (Factor 1) as the dependent variable, used the city where the clinical practice of the physician is located (city of clinic), and whether the physician indicated television (TV) as a source of ASD knowledge as the independent variables. This model had statistically significant additive associations with the factor (*P*_City of Clinic_ < 0.01 & *P*_TV_ < 0.01). Physicians whose practice was in the city of Brăila had the lowest level of knowledge regarding the stigma about ASD in Romania compared to physicians whose practice was in the Capital, Bucharest (AMS: 0.29 vs. 1.93; *P* < 0.01). Similarly, physicians who practiced in other smaller cities were also less knowledgeable about the stigma associated with ASD compared to physicians whose medical practice was in Bucharest (AMS: 1.44 vs. 1.93; *P* < 0.01). Further analysis of the clinic location and television (TV) as source of ASD knowledge among physicians revealed that a significant interaction between these two variables (*P* = 0.02) in relation to physicians’ knowledge of stigma related to ASD. Specifically, among physicians who indicated that they had heard of ASD from TV, the physicians whose clinic was located in Bucharest had the highest knowledge of stigma related to ASD (TV-Yes AMS: Bucharest: 2.39 vs. Other: 1.58 vs. Braila: 0.71 vs. Suçeava: 0.60). Among physicians who indicated that they had not heard of ASD from TV, physicians whose clinics was located in Suçeava had the highest knowledge of stigma related to ASD (TV-No AMS: Suçeava: 2.45 vs. Bucharest: 1.64 vs. Other: 1.59 vs. Braila: 0.0001).

### Characteristics associated with physicians’ knowledge of potential cause(s) of ASD (factor 2)

The GLM with the “physicians’ knowledge regarding potential causes of ASD” sub-score (Factor 2) as the dependent variable determined that physicians’ age, and the average number of patients seen daily were significantly associated with this factor (*P*_Age_ < 0.01 & *P*_Average # of Patients seen daily_ < 0.01). We found that younger physicians (i.e., age ≤ 35 years) were more knowledgeable about potential causes of ASD than the physicians over 35 years of age (AMS: 2.90 vs. 2.18, *P* < 0.01). Physicians who had a larger clinical practice (i.e., they saw, on average, more than 40 patients daily) had a higher level of knowledge about potential causes of ASD than physicians who saw an average of 20 patients or less daily (AMS: 3.10 vs. 2.11, *P* = 0.04). We also checked for potential interactive effects between these two variables but did not find any significant interactions in relation to knowledge of potential cause(s) of ASD.

### Characteristics associated with physicians’ knowledge of the behavior of children with ASD (factor 3)

The multivariable GLM with the “physicians’ knowledge of the behavior of children with ASD” sub-score (Factor 3) as the dependent variable identified that physicians’ age (*P* = 0.02), city of clinic (*P* < 0.01), and whether the physician attended a public or private medical school (*P* = 0.03) were significantly associated with Factor 3. Older physicians (> 35 years of age) were more knowledgeable about the behavior of children with ASD compared to younger physicians (AMS: 0.64 vs. 0.37, *P* = 0.02). Physicians who attended a public medical school were more knowledgeable about the behavior of children with ASD than those who attended a private medical school (AMS: 1.02 vs. 0.57, *P* = 0.03). In addition, physicians whose clinical practice was in Suçeava were more knowledgeable about the behavior of children with ASD than the physicians who practiced in Bucharest (AMS: 0.30 vs. 0.99, *P* < 0.01). We found that the interactive effects between these three variables were not significant in relation to physicians’ knowledge of the behavior of children with ASD.

### Characteristics associated with physicians’ knowledge of misconceptions about ASD (factor 4)

Multivariable GLM analysis for Factor 4, physician’s knowledge of misconceptions regarding ASD, revealed that the following SAKs had an additive effect with the factor: TV (*P* < 0.01) and radio (*P* = 0.01). However, to avoid multicollinearity due to a high correlation between these two variables, we defined a new variable for physicians that responded “Yes” to either of these two variables. Physicians that heard of ASD from either the TV or the radio were less knowledgeable about misconceptions regarding ASD than physicians who did not, however this association was marginally significant (AMS: 0.51 vs. 0.62; *P* = 0.09).

### Characteristics associated with physicians’ knowledge of special education needs of children with ASD (factor 5)

Our multivariable GLM with the “physicians’ knowledge regarding special education needs of children with ASD” sub-score as the dependent variable only identified one additive association: physicians who learned of ASD from newspapers were more knowledgeable regarding the special needs of children with ASD than physicians that did not (AMS: 1.00 vs. 0.94, *P* < 0.05).

### Characteristics associated with physicians’ overall knowledge of ASD (Total score)

Our multivariable GLM models with total ASD score of “physicians’ knowledge of ASD” as the dependent variable identified that physicians with experience in Pediatrics (Model 1), or Psychiatry (Model 2), or both ward rotations (Model 3) at medical school had no significantly different AMS than those without these experiences (all *P* > 0.75), though physicians with continuing medical education as a source of ASD knowledge had significantly higher AMS than those without continuing medical education (*P* < 0.01). Other associations regarding physicians’ knowledge of ASD with other ward rotations are reported in Table 5.

**Table 5:** Characteristics associated with Physicians’ ASD knowledge related to the five sub-scores from each factor and total score based on final Multivariable General Linear Models (GLMs) along with the adjusted means of the five sub-scores from each factor and total score for each level of the independent variables in GLMs

Factor		Variable Name	Categories	Adjusted Mean Sub-Score (AMS)†	Adj. Mean difference^*^	***P*** value^**^
**1 - Knowledge of stigma related to ASD (*N*** = 364)^***^		City where the clinic is located	Bucharest (Reference)	1.93	–	–
			Brăila	0.29	−1.64	< 0.01
			Suçeava	1.98	0.04	0.86
			Other	1.44	−0.50	< 0.01
		Television as a source of ASD knowledge	Yes	1.70	0.34	0.02
			No (Reference)	1.38	–	–
**2 - Knowledge of potential cause (s) for ASD (*N*** **= 341)**		Age of physician at time of survey (years)	≤ 35 years old (Y/O)	2.90	0.72	< 0.01
			> 35 Y/O (Reference)	2.18	–	–
		Average number of patients seen in a day	0–20	2.11	−0.98	0.04
			21–40	2.42	−0.68	0.25
			≥ 41 (Reference)	3.10	–	–
**3 - Knowledge of the behavior of children with ASD (*N*** **= 350)**		Age of physician at time of survey (years)	≤ 35 years old (Y/O)	0.37	−0.26	0.02
			> 35 Y/O (Reference)	0.64	–	–
		Type of medical school attended	Private	0.57	−0.57	0.03
			Public (Reference)	1.02	–	–
		City where the clinic is located	Bucharest (Reference)	0.99	–	–
			Braila	0.95	0.09	0.84
			Suceava	0.30	−0.69	< 0.01
			Other	0.94	−0.07	0.61
**4 - Knowledge of misconceptions about ASD (N = 377)**		Television or radio as a source of ASD knowledge	Yes	0.51	−0.11	0.09
			No (Reference)	0.62	–	–
**5 - Knowledge of educational needs regarding children with ASD (N = 379)**		Newspaper as a source of ASD knowledge	Yes	1.00	0.06	0.05
			No (Reference)	0.94	–	–
**Total Score (N = 371)**	**Model 1**	Ward rotation: Psychiatry	Yes	6.40	−0.03	0.92
			No (Reference)	6.43	–	–
		Continuing education as a source of ASD knowledge	Yes	6.81	0.78	< 0.01
			No (Reference)	6.03	–	–
	**Model 2**	Ward rotation: Pediatrics	Yes	6.40	−0.15	0.75
			No (Reference)	6.55	–	–
		Continuing education as a source of ASD knowledge	Yes	6.86	0.78	< 0.01
			No (Reference)	6.08	–	–
	**Model 3**	Ward rotation: Psychiatry and Pediatrics	Yes	6.41	0.03	0.93
			No (Reference)	6.39	–	–
		Continuing education as a source of ASD knowledge	Yes	6.79	0.77	< 0.01
			No (Reference)	6.01	–	–

†Least Squared Means; ^*^Adj. Mean difference from the reference; ^**^H_0_: Least Square Mean (LSMean) (group 1) = LSMean (group 2); ^***^Interactive effect of ‘city where the clinic is located’ and ‘television as a source of ASD knowledge’: *P* < 0.01

## Discussion

To our knowledge, this is not only the first study that assessed the knowledge of physicians in Romania about ASD, but it is also one of the few studies that used factor analysis to define latent constructs (domains) and established their validity. A few other studies have also established internal consistency of their questionnaires that assessed competency of medical students in medical schools in relation to their knowledge about ASD [21]. In our study we used factor analysis to identify five independent domains that describe different aspects of the knowledge of Romanian physicians about ASD. In the following, we will discuss our findings in the context of each of these important issues.

### Developing latent domains for knowledge of Romanian physicians about ASD

It is customary to consider a single composite score to characterize the physicians’ knowledge about a disease or a disorder. However, for complex disorders such as ASD, there may be interest in assessing knowledge of physicians regarding different aspects of ASD that cannot be characterized meaningfully into a single composite score. Since the 12 ASD knowledge questions in this survey sought information about different aspects of physicians’ knowledge about ASD, we initially used factor analysis [31], to determine the underlying latent factors. Based on the inclusion criteria of an Eigenvalue ≥1.00 for the composite sub-scores we identified five independent composite sub-scores: stigma, potential causes, children’s behavior, misconceptions, and educational needs associated with ASD. These five composite sub-scores explained nearly 64% of the total variance between the 12 knowledge questions included in the factor analysis. Though some may aim to explain a higher percentage of the total variance in the 12 knowledge questions by extracting a higher number of composite sub-scores (i.e., > 5), one should consider the trade-off between a higher number of factors extracted and challenges in the interpretation of additional factors in the context of sub-domains of ASD knowledge in Romanian physicians. This is particularly important if this leads to extracting additional factors with Eigenvalues < 1, meaning that the value of that one additional factor in explaining the total variance is less than that of one variable (i.e., one question among the 12 questions).

It is important to keep in mind that in this kind of situation a single overall knowledge score may not be meaningful because the underlying factors in the scale could negate each other’s scores, making it very difficult to interpret. Under such situations, identification of underlying sub-constructs may be useful for educational purposes, as this uncovers independent domains of medical knowledge of physicians. Investigating the associations between physicians’ characteristics and certain domains of knowledge are useful in building educational strategies to target those domains.

### The overall score as the perceived competence of practicing physicians in Romania about ASD

Although the findings from our descriptive analysis indicate that the Romanian physicians are knowledgeable about basic issues related to ASD, the overall responses from different parts of the survey suggest that majority of the Romanian physicians may benefit from additional training or continuing medical education in certain aspects of ASD, including assessments and causes of ASD. In the following, we discuss these issues.

### Findings from descriptive analysis related to knowledge of Romanian physicians about ASD

Findings from our survey indicate that the majority of physicians in Romania have some basic knowledge about ASD. For example, about 73% of physicians correctly responded to an important characteristic of children with ASD: being detached from family members. Additionally, about 94% of the physicians were aware of the special educational needs for children with ASD. Surprisingly, 92% of physicians thought that children with ASD could grow out of this complex disorder. Though cognitive behavioral therapy may help a large portion of children with ASD improve their symptoms and quality of life, most children do not grow out of the spectrum since there is still no cure for ASD [33]. Additionally, physicians’ responses to various questions in the survey indicate a deficiency in knowledge about some aspects of ASD which could be improved by relevant supplementary educational activities. For example, since only 16.3% of physicians correctly responded to all questions regarding the potential causes of ASD, we suggest provision of additional continuing education courses focused on improving physician’s knowledge related to potential causes of ASD.

Similar to our survey, a study of 93 Youth and Family Center physicians from the Netherlands that assessed the physicians’ knowledge about ASD, and the stigmatizing attitude they held towards ASD and mental illness found that the physicians had sufficient general knowledge about ASD [34]. However, physicians in this study still had limited knowledge in specific areas such as language and communication related issues in ASD patients.

### Characteristics associated with physicians’ overall knowledge of ASD (Total score)

While it may be easier to assume that the knowledge score about ASD is additive, further analysis of interactive relationships may help further clarify relationships between factors associated with the sub-scores. The total score did not show any significant associations with the same variables that were significantly associated with the five sub-scores. While the total score was significantly associated with attending the Emergency ward rotation (*P* = 0.02), and continuing medical education as a source of ASD knowledge (*P* < 0.01), multivariable GLM results for each sub-score did not show any associations with these variables. However, attending the Psychiatry and Pediatrics ward rotations at medical school did not have any significant associations with the overall knowledge of physicians on ASD (all *P* > 0.75). Although the postgraduate curriculum added the requirement of a 1 month pediatric psychiatry rotation for family physicians in the residency program, as well as 3 months of pediatric psychiatry training, our findings suggest that the content of training may not provide sufficient education on neurodevelopment disorders such as ASD. Considering that nearly all of the participants in this survey were trained in Romania, these findings support the need for additional modification of curriculum on neurodevelopmental disorders including ASD in Romanian medical schools.

Findings from GLMs using the total score as the outcome variable did not reveal any significant characteristics associated with those found in sub-scores, except for experience in the Emergency ward rotation, and continuing education as a source of ASD knowledge. This important observation indicates the possibility that the sum of the 12 knowledge scores may not be meaningful because the underlying factors in the scale could negate each other’s scores, making it very difficult to interpret. However, the sub-scores are constructed in a manner that they provide independent information regarding different aspects of ASD knowledge.

### Characteristics associated with knowledge of physicians related to each of the five domains

#### Characteristics associated with physicians’ knowledge of stigma related to ASD (factor 1)

Our findings related to the effects of the location of the physicians’ clinic, and whether or not they learned of ASD through TV on physicians’ knowledge about stigma related to ASD in Romania, indicate that physicians from Brăila and other smaller cities were less aware of stigma associated with ASD than physicians from Bucharest (AMS difference = − 1.64 for Braila vs. Bucharest; AMS difference = − 0.50 for Other cities vs. Bucharest). Physicians who did hear of ASD through TV were more knowledgeable about the stigma related to ASD than physicians who did not (AMS difference = 0.34 for TV-Yes vs. TV-No). Considering the role that media and television play in our modern society, the varying portrayals of ASD in news and other media may contribute to the misconceptions and stigma related to ASD. Studies related to other disorders describe increased stigma and misconceptions of mental health due to their portrayals on TV [35–39]. However, few studies assessed this relationship in physicians, and no studies that assessed stigma and perceptions of ASD through television in physicians or other populations were found.

The significant interactive association between city of clinic and television (TV) as source of ASD knowledge among physicians in regards to physicians’ knowledge of stigma related to ASD (*P* < 0.01) shows the necessity of searching for complex interpretations in the interactive effects among physicians’ characteristics, specifically in regards to underlying domains of knowledge on ASD among physicians. Physicians’ knowledge of stigma related to ASD under the model with interactive effects revealed additional information that the model with only the additive effects could not provide. Specifically, this interaction indicates that the association of TV as a source of ASD knowledge with physicians’ knowledge of stigma related to ASD is dependent on which city the physician’s clinic is located.

#### Characteristics associated with physicians’ knowledge of potential cause(s) of ASD (factor 2)

Our findings from analysis of Factor 2 indicate that younger physicians (≤ 35 years old) were more aware about the potential causes of ASD (AMS difference = 0.72). Similarly, a survey of 348 general physicians in Pakistan reported that physicians less than 30 years of age were more knowledgeable about ASD than their older counterparts [13], and a study of 313 family medicine residents in Turkey also reported that physicians over the age of 35 were less knowledgeable about ASD than the younger residents [14]. This finding may be a result of the continuously expanding pool of research surrounding ASD, and the inclusion of new findings in school curricula as new knowledge is uncovered. However, this finding also suggests that more efforts are needed to bring awareness about the potential causes of ASD to older physicians through formal continuing education programs. Additionally, physicians who saw 20 or less patients in a day were significantly less knowledgeable about potential causes of ASD than physicians who saw more than 40 patients in a day (AMS difference: − 0.99), as were physicians that saw between 21 and 40 patients per day (AMS difference: − 0.68). Physicians’ general knowledge about various disorders including ASD may also increase as medical knowledge expands, and physicians’ practices grow and they come across patients with a variety of ailments [40].

#### Characteristics associated with physicians’ knowledge of the behavior of children with ASD (factor 3)

Older physicians (> 35 years) were more knowledgeable about the behavior of children with ASD than younger physicians (AMS difference = − 0.26). Physicians who attended a public medical school were more knowledgeable about the behaviors of children with ASD private school counterparts (AMS difference = − 0.57). Experience dealing with children with ASD gained over the years of their practice may provide physicians with a better understanding of the behaviors displayed by ASD children. Additionally, physicians with clinics in Suceava were significantly less knowledgeable than their colleagues that had clinics in Bucharest about the behavior of children with ASD (AMS difference = − 0.68). Since larger cities, like the Romanian capital of Bucharest, usually house many of the larger educational institutes in the country compared to the smaller cities, physicians from larger cities may have access to more resources and the most up-to-date information about the behavior of children with ASD. Physicians from larger cities may also have more knowledge of this factor as they may be more likely to be exposed to children with ASD than physicians in smaller cities.

#### Characteristics associated with the physicians’ knowledge of misconceptions about ASD (factor 4)

Though we analyzed various variables, using GLM models to determine characteristics associated with physicians’ knowledge of misconceptions regarding ASD, we found no significant additive or interactive effects. Even though there were no associations found using basic GLM analyses, future exploratory analyses may be needed to assess the relationship between/among these variables.

#### Characteristics associated with physicians’ knowledge of educational needs of children with ASD (factor 5)

We also found that whether or not physicians learned of ASD through the newspaper had an additive effect with physicians’ knowledge of educational needs of children with ASD. Physicians who did not learn of ASD through the newspaper (Newspaper-No) were less aware of the special educational needs of children with ASD compared to the reference group of physicians who had heard of ASD through the newspaper (AMS difference = 0.06; Newspaper-No vs. Newspaper-Yes, *P* = 0.05). Newspapers, including ones published online, are still important avenues of information for the general public. Pesonen et al. analyzed 210 full-length newspaper articles in Finland published between 1990 and 2016 to qualitatively assess how articles present ASD to the public. The authors found that 110 out of 210 articles reported information in simple and concise clinical language to educate the public about ASD. Though this study does not assess levels of knowledge for any population, we can see through this paper that newspapers can be a good source of ASD knowledge as long as readers keep in mind that the articles may present ASD knowledge with some bias [41].

Overall, it is necessary to assess the knowledge, attitude, and practices of physicians about ASD as their views and background regarding ASD will potentially affect their practice and their diagnosis of children with ASD. Greater knowledge of ASD is essential for physicians for various reasons such as better service delivery [42, 43] for ASD children, and earlier identification of cases for appropriate therapies [13] that rely heavily on the physician’s knowledge of ASD. Additionally, greater knowledge of ASD among physicians can also increase the trust between parents, patients, and their physician [17]. Furthermore, as knowledge increases among physicians, they will be able to better educate future generations of physicians. As a result, this may help to decrease the concerns amongst medical students in pursuing psychiatry or psychology, thus potentially increasing the number of students specializing in that field, further benefitting the Romanian population as well.

We provided evidence that it is important to use validity of the construct and internal consistency to assess the knowledge, attitude, and practices of physicians, which other studies [20, 21] also suggested. We also showed that using the sum of the Likert-scale scores, or reporting percentage of responses to questions, may not help to obtain an easier interpretation. Therefore, we highly recommend that in future surveys investigators use similar methods, like factor analysis, to identify or confirm the validity of constructs before creating a total score for such analyses. Furthermore, we recommend exploring possible interactions between independent variables in relation to other knowledge sub-scores to determine variables that may have synergistic effects for a more precise understanding of the related issues influencing physicians’ knowledge, attitude, and practices in the context of a complex disorder such as ASD.

## Limitations

We acknowledge that for this study we did not select a random sample of physicians who could represent all physicians in Romania. Instead, data were collected by the team of volunteers from the physicians’ offices in Romania, as described in the methods section. Hence our samples in this study may not be representative of the population of physicians practicing in Romania. Since physicians that are interested in ASD are more likely to participate in the study, the responses may be skewed towards physicians that had existing knowledge of ASD. The results may also be influenced by the non-response bias for some questions. For example, for the question asking whether they have heard of ASD, those who answered “Yes” were identified but the remaining answers were left blank. Therefore, for some limited number of variables it is not clear whether the response to the remaining questions is “No,” or something other than these two responses. Additionally, due to the nature of some of the questions, such as the practitioner’s year of graduation, the study may also have been affected by recall bias. Our study was also dependent on self-reported data regarding knowledge, practice, and attitude of physicians in Romania regrading ASD. Moreover, we acknowledge that we have not performed any confirmatory factor analysis to establish the stability of the associations reported in this study. We further acknowledge that in the past two decades the definition and classification of Autism Spectrum Disorder (ASD) has changed in the US [44, 45]. Based on the latest disease classification (DSM-5), the term “ASDs” has been replaced by “ASD”. Since the word “Autism” is used interchangeably with the term “ASD” by a large portion of physicians, in this study for simplicity in communication we have considered the terms Autism, ASD, and ASDs as the same.

## Conclusions

In this study, we established construct validity and characterized five latent domains related to Romanian physicians’ knowledge about ASD that include stigma, potential causes, behavior in ASD children, special education needs, and misconceptions related to ASD using factor analysis. Though practicing physicians in Romania have adequate general knowledge about ASD, greater focus is needed on increased awareness of stigma and misconception surrounding this disorder, as well as its symptoms and potential causes. Since the sub-scores provide independent information regarding different aspects of ASD knowledge overall, it is recommended that future studies of this kind use factor analysis to identify independent sub-scores before using the total score. However, a lack of a significant association of the knowledge of physicians on ASD with neither the Psychiatry, nor the Pediatric ward rotations at medical school may support the need for improving the curriculum on ASD in Romanian medical schools. Furthermore, we recommend that future studies that intend to identify predictors of physicians’ knowledge regarding any disorders, including ASD, also explore potential interactive effects of the specific characteristics in addition to their additive effects.

## Supplementary Information

**Additional file 1:.**

## Data Availability

The datasets used for the current survey will not be publicly available due to possible identification of the individual sources or the location of physicians’ practice. However, these data are fully secured and could become available under certain circumstances from the corresponding author based on a reasonable and a justified request.

## Abbreviations

ADHD: Attention Deficit Hyperactivity Disorder
ADI-R: Autism Diagnostic Instrument-Revised
ADOS: Autism Diagnostic Observation Schedule
ADOS-2: Autism Diagnostic Observation Schedule-2
AMS: Adjusted Mean Score
ASD: Autism Spectrum Disorder
ASRS: Autism Spectrum Rating Scale
Carol Davila-UMF: “Carol Davila” University of Medicine and Pharmacy
CHAT: Checklist for Autism in Toddlers
CS-TSA: Chestionarul de Screening pentru Tulburări de Spectru Autist
DSM-5: Diagnostic and Statistical Manual, Fifth Edition
ERC: Ethical Review Committee
GLMs: General Linear Models
IRBs: Institutional Review Boards
KAP: Knowledge, Attitudes, and Practices
LSMeans: Least Square Means
REDCap: Research Electronic Data Capture
SCQ: Social Communication Questionnaire
SAK: Source of ASD knowledge
SEN: Special Education Needs
UMF: University of Medicine and Pharmacy
UTHealth: University of Texas Health Science Center at Houston
