# Supplementary material for: Construct validity for the self-reported competency and sub-construct associated characteristics of Romanian physicians in autism spectrum disorder
Source: BMC Med Educ. 2021 Nov 20;21:588. doi: 10.1186/s12909-021-02999-9 (PMC8605522; doi:10.1186/s12909-021-02999-9)
Supplement: Supplementary file 1 — Additional file 1:. [file 12909_2021_2999_MOESM1_ESM.pdf]

Supplementary Material

Construct validity for the self-reported competency of Romanian physicians in Autism Spectrum Disorder, and physicians' characteristics associated with these sub-constructs

Mohammad H. Rahbar [1,2,3 *], **Iuliana Dobrescu** [4,5], **Shezeen Gillani** [3], **Manouchehr Hessabi** [2], **Sori Kim** [2, 6], **Mihaela Stancu** [4,5], **Florina Rad** [4,5]

[1] Division of Clinical and Translational Sciences, Department of Internal Medicine, McGovern Medical School, The University of Texas Health Science Center at Houston, Houston, Texas, USA.

[2] Biostatistics/Epidemiology/Research Design (BERD) core, Center for Clinical and Translational Sciences (CCTS), The University of Texas Health Science Center at Houston, Houston, Texas, USA.

[3] Department of Epidemiology, Human Genetics, and Environmental Sciences, School of Public Health, The University of Texas Health Science Center at Houston, Houston, Texas, USA.

[4] Child and Adolescent Psychiatry Department, University of Medicine and Pharmacy "Carol Davila", Bucharest, Romania.

[5] Child and Adolescent Psychiatry, "Prof. Dr.Alex. Obregia" Psychiatry Hospital, Bucharest, Romania.

[6] Department of Biostatistics & Data Science, School of Public Health, The University of Texas Health Science Center at Houston, Houston, Texas, USA.

*** Corresponding Author:** Mohammad H. Rahbar, PhD, 6410 Fannin Street, Suite 1100.05, UT Professional Building, Houston, TX 77030, USA. Phone: (713)500-7901. Fax: (713)500-0766. Email: Mohammad.H.Rahbar@uth.tmc.edu

MINISTRY OF HEALTH OF ROMANIA

PUBLIC HEALTH HUMAN RESOURCES CENTER

TRAINING CURRICULUM IN THE SPECIALTY

PEDIATRICS

All rights regarding the publication and dissemination of this paper belong to the Ministry of Health

MINISTRY OF HEALTH OF ROMANIA

2017

Google Translation of:

TRAINING CURRICULUM IN THE SPECIALTY PEDIATRICS

Definition of specialty:

Pediatrics is the specialty that deals with the medical pathology of the children from 0 to 18 years of age and it includes a prophylactic field (puericulture) and a curative field, to which is associated the additional study of some subfields which constitute specialties derived from pediatrics, as follows:

1. Pediatric cardiology.
2. Pediatric gastroenterology.
3. Pediatric hematology and oncology.
4. Pediatric Pneumology (Pulmonology).
5. Pediatric nephrology.

Graduates of the specialties listed under points 1 to 5 will be certified as “pediatric medical specialists' and (follows the derived specialty)..........” having the knowledge and qualifications to perform on-call duty in pediatric specialty and to provide pediatric care in accordance with the skills acquired according to the training curriculum.

The curriculum provides a number of 200 teaching hours (lecture, seminar, case presentations, and demonstrations) per year of university study, for the topic presented, and 40 to 50 hours of individual study. Training through the common core takes 3 years. After graduating the common core, for General Pediatrics, respectively for each derived specialty, specific training will continue in accordance to the curricula drawn up separately.

The residency program coordinator is nominated by the medical higher education institution and is having the role of coordinating the process of the residency program and choosing - for each clinical ward - a responsible doctor for residency training. The head of residency training, in collaboration with the head of department, assigns the residents to the specialist/primary doctors in the department who are to become guiding doctors and who will carry out the training of residents in accordance with the approved curriculum. During the on-call period, the specialist/primary on-call physician becomes a guide in clinical activity for the duration of the call.

The program coordinator will monitor compliance with the duration and curriculum of each training module. Within the same year of training, the residency program coordinator will ensure the rotation of

resident doctors so that they complete all the modules for the year of study. It is permitted to take one module in a split mode in order to avoid overcrowding of some wards and the simultaneous allocation of a large number of residents to one medical supervisor. The training modules will be carried out with priority in the pediatric wards where there is a teaching staff (full or associate) with a specific specialty/ subspecialty/status training module; if there are no tutors in any of the pediatric clinical wards in specialty of the module, the traineeship will be carried out in the adult specialty clinic.

The person in charge of the residency training verifies and is responsible for the completion of the topics in the training curriculum, organizes the educational activity of the residents at the department level, assigns the residents to mentors. The allocation of residents will be done in accordance with the professional competences of the doctors (specialty, subspecialty, and certification) and the specific training module. The person responsible for the residency training organizes and participates in the evaluation of residents at the end of each module.

Module 4 - Pediatric Psychiatry:

No. of course hours: 30

No. of practical hours: 300

I. Thematic studies courses

1. Examination of the main mental functions according to age in children and adolescents.
2. Stages of normal cognitive and emotional development. Developmental disturbances at different ages: toddler (affect seizures, crying spasm), school child (effect of excessive use of TV technology, computer, phone; aggression; bullying phenomenon), adolescent (inappropriate behavior, drug and alcohol abuse).
3. Intellectual disability.
4. Sleep disorders in children and adolescents.
5. Eating disorders.
6. Anxiety disorders in children and adolescents.
7. Depression and suicidal risk.
8. Autism spectrum disorders.
9. Child and adolescent abuse.
10. Psychotic disorders.
11. Mental disorders in somatic diseases.
12. Counselling and parenting techniques used in child and adolescent psychiatry.

II. Practical activities

1. Application of screening tools for the detection of the main mental disorders in children and adolescents.
2. Carrying out the medical history and mental status examination to identify both the cognitive and emotional developmental problems.
3. Identifying risk factors associated with the development or worsening of the main mental disorders in children and adolescents.
4. Identifying suicidal risk and adopting the first measures of the intervention plan in suicidal crisis.
5. Drawing up and communicating to the child/adolescent and the family the psychosocial plan according to age, pathology and identified risk factors.

6. Drawing up and communicating to parents the plan of parenting techniques to be used according to the child's age and/or pathology.

III. Specific objectives

- To become familiar with the general concepts of anamnesis and mental status examination of children and adolescents.
- Screening and identification of the main cognitive and/or emotional developmental problems in children and adolescents.
- Screening and identification of main psychiatric disorders in children and adolescents.
- Familiarization with the concepts of psych hygiene and parenting.

IV. Expected results

- To be able to identify cognitive and/or emotional developmental problems in children and adolescents.
- To be able to recognize the main signs of mental disorders in children and adolescents.
- To identify and correctly access medical services where they can refer patients for confirmation of psychiatric diagnosis and specialist intervention.
- To identify the patient at risk of suicide and apply first intervention measures in suicidal crisis.
- To correctly communicate to the child/adolescent/family the necessary psychosocial measures and parenting techniques.

GENERAL EDUCATIONAL AIMS AND OBJECTIVES:

The general objectives of the common core of training in pediatrics include the acquisition of knowledge, abilities and skills in the following areas:

I. Communication;
II. Ethics and professionalism;
III. Quality assurance of medical care;
IV. Pediatric expertise (clinical and procedural skills and interpretation of investigations);
V. Organization and management.

I. Communication.

The resident must acquire the following skills:

A. Communication with the patient and caregivers:

- Ability to appropriately communicate with patients and caregivers based on socioeconomic level and cultural level, including communicating bad news;
- Ability to retrieve information conveyed through verbal and non-verbal language by the patient and/or caregivers (relationship between child and caregivers, warning signs for conflicting elements in patient's socio-familial environment, etc.);
- Ability to adapt medical care to the socio-cultural and educational particularities of the patient and his/her family for the benefit of the sick child;
- Ability to resolve conflicts between patients/carvers and members of the medical team;
- Use of appropriate methods to inform the patient and his/her family about adverse effects of the medical care (including iatrogenicity).

B. Communication with other health professionals:

- Ability to communicate synthetically and clearly within the medical or multidisciplinary team;
- Recognizing and respecting the role, responsibilities and competence of other medical team members;
- Ability to resolve conflicts within the health care team;
- Ability to communicate effectively and succinctly with other individuals and institutions in the health system;
- Ability to present medical information in a succinctly way while discussing with medical staff in within the on-call report, case presentations, etc.;
- Ability to collaborate with colleagues from other medical specialties in order to facilitate transition of the chronically ill adolescent into the adult patient's medical network.

C. Communication with institutions outside the medical system:

- Ability to collaborate with teaching staff, social workers and caregivers for prophylactic and therapeutic actions to ensure the child's health;
- Ability to synthetically draft documents for forensic institutions that provide interventions related to the medical act itself.

II. Ethics and professionalism.

The resident must acquire the following skills:

- Knowledge and compliance of legal provisions relating to the practice of medicine in Romania, including the Code of Ethics of the physician;
- Knowledge and compliance with the legislation on patients' rights, including compliance with confidentiality and adopting a non-discriminatory attitude regardless of age, gender, religion, ethnic origin and disability;
- Knowledge and compliance with the internal rules and regulations of the hospital where they work; knowledge of the administrative structure of the hospital and the working relationship between the hospital's staff;
- Ability to provide the highest quality of medical care and maintain high standards through continuing medical education;
- Knowledge and application of local and national diagnostic and treatment protocols; use of the results of scientific research in medical practice;
- Compliance with legal and moral obligations to report illnesses or situations of potential or actual abuse;
- Ability to present clinical cases or lectures;
- Ability to evaluate and present the content of scientific meetings;
- Ability to access medical information, understand the difference between research and clinical audit;
- Ability to use and understand the interpretation of simple statistical tests;
- Ability to study medical literature for evidence;
- Mastering the methodology of scientific research.

III. Ensuring the quality of healthcare.

The resident must acquire the ability to:

- Synthetic knowledge of the organization of the quality structures in the hospital where he/she works;
- Knowledge of the tools for improving the quality of medical care;
- Identification of the main categories of medical errors;
- Identification of situations with increased risk for adverse events in the medical activity of child care;
- Identification and reporting of adverse events/effects of medical activity;
- Knowledge of the basic elements in conducting a clinical audit;
- Knowledge of appropriate methods applicable to reduce adverse events;
- Understanding the principles, methods and tools used to increase patient safety.

IV. Pediatric expertise.

The resident must:

- Possess domain-specific knowledge and skills: clinical evaluation, recognizing a seriously ill patient and initiation of emergency measures, formulation of a positive and differential diagnosis, preparation of the investigation and therapeutic plan, modifying the plan according to the clinical evolution and paraclinical results;
- Learn how to complete medical documents;
- Acquire knowledge of the child's social vulnerability and the protective measures required in such situations.

V. Organization and management.

The resident must acquire the following skills:

- Rational use of time;
- prioritizing activities and tasks;
- Delegation of tasks;
- Identifying and controlling stressful situations and taking action to minimize their effects;
- Controlling crisis situations caused by lack of resources;
- Cultivating the ability to identify and learn from one's mistakes;
- Recognizing personal and professional limits;
- Drafting medical documents (medical report, discharge note, legal documents) in a way that is understandable, legible and at time.
